# Role of rapidly evolving immunotherapy in chronic active Epstein-Barr virus disease

**DOI:** 10.3389/fimmu.2024.1451977

**Published:** 2024-12-03

**Authors:** Sijia Yan, Xi Ming, Xiaojian Zhu, Yi Xiao

**Affiliations:** Department of Hematology, Tongji Hospital, Tongji Medical College, Huazhong University of Science and Technology, Wuhan, Hubei, China

**Keywords:** chronic active Epstein-Barr virus disease, Epstein-Barr virus, immunotherapy, PD-1 inhibitor, Epstein-Barr virus specific cytotoxic T lymphocytes

## Abstract

Chronic active Epstein-Barr Virus disease is a kind of Epstein-Barr Virus associated T/NK cell lymphoproliferative disease. At present, there is still a lack of standard therapeutic regimen for its treatment, but its basic treatment principles include controlling inflammatory response, anti-tumor proliferation, and immune reconstitution. Hematopoietic stem cell transplantation is currently the only method that can cure this disease. In recent years, immunotherapy has developed rapidly and is widely used in the treatment of various hematological malignancies; various immunotherapy drugs, including PD-1 inhibitors, have also demonstrated their safety and efficacy in CAEBV, while immune cell therapies such as Epstein- Barr virus-specific T cells have also displayed their unique advantages in CAEBV.

## 1 Introduction

**Figure 1:**
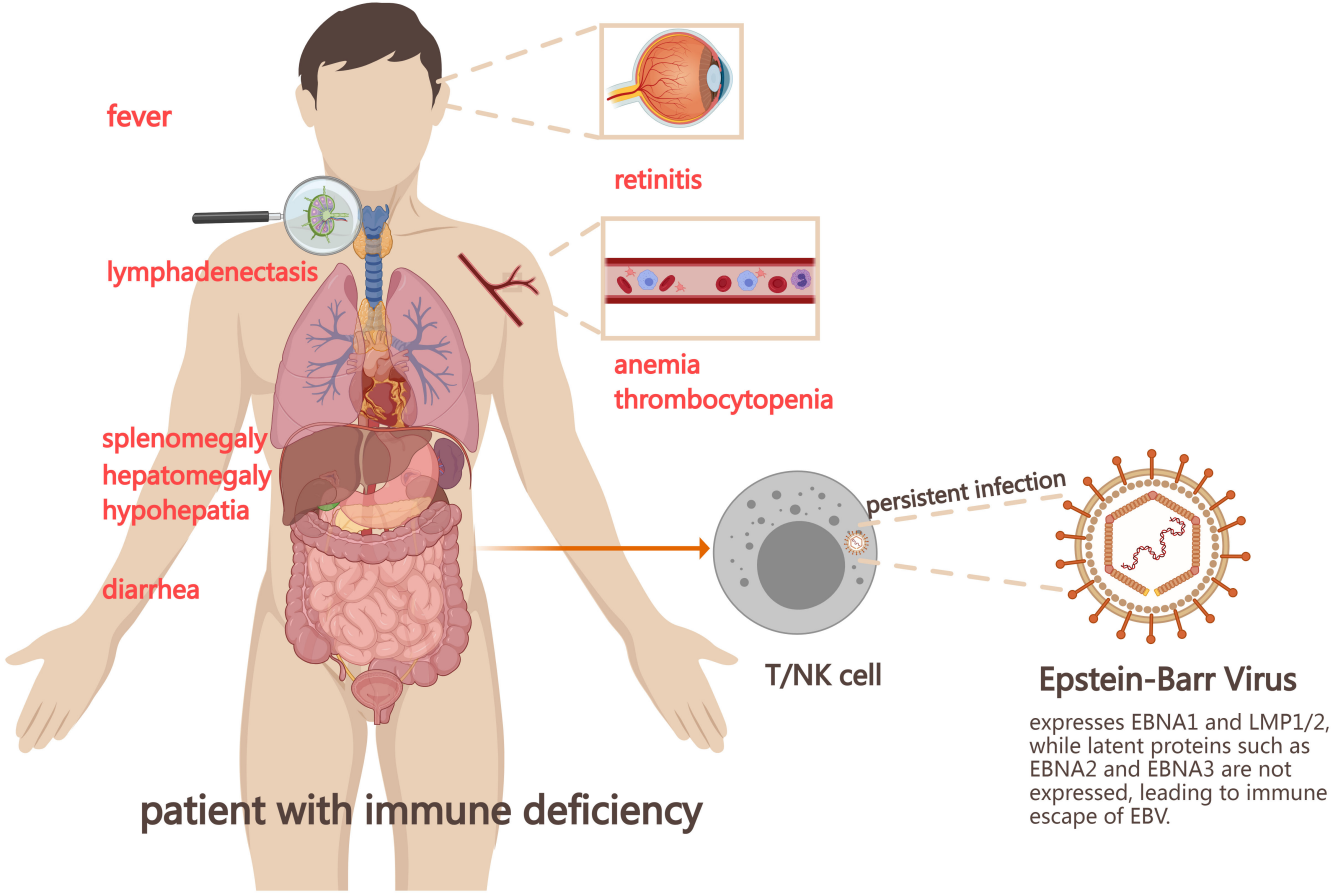
Mechanism and symptoms of chronic active Epstein-Barr virus disease (CAEBV). EBV infects the human body, forming the latent infection in the body. When the immune function of the body is impaired or the virus replication is enhanced, it leads to a variety of EBV positive lymphocyte proliferative diseases, including CAEBV. EBV, Epstein-Barr virus; NK cell, natural killer cell; EBNA1, Epstein-Barr virus nuclear antigen1; LMP, latent membrane protein.

The Epstein-Barr virus (EBV) is an extremely common herpes virus affecting 90% of the global population. It was the first confirmed oncovirus associated with various tumors and lymphoproliferative diseases such as nasopharyngeal carcinoma, Burkitt’s lymphoma, Hodgkin’s lymphoma, and gastric cancer (1). Primary EBV infection first infects naive B cells and establishes latent infection. The imbalance between the body’s immune response and the replication of EBV can lead to a variety of EBV-positive lymphocyte proliferative diseases involving B, T, and NK cell (2). Chronic active Epstein Barr Virus disease (CAEBV) is a lymphoproliferative disease caused by EBV infection of T or natural killer cells, characterized by persistent or recurrent infectious mononucleosis-like (IM)-like symptoms, including fever, hepatosplenomegaly, and enlargement of lymph nodes. Some patients present with invasive course and serious complications. Poor prognosis is associated with hemophagocytic lymphohistiocytosis (HLH), multiple organ failure, and progression to leukemia/lymphoma, with poor prognosis (**Figure 1**) (3–5). In 2016, the World Health Organization classified severe CAEBV as EBV-associated T/NK cell lymphoproliferative disease (EBV-T/NK LPD).

But its pathogenesis remains unclear. Latent gene EBV-encoded small RNA (EBER)1 and BamHI-A rightward transcripts(BART) were detected in all CAEBV patients, and latent membrane protein (LMP) 2 was found in most patients. However, the levels of EBV nuclear antigens (EBNA) 1 and LMP1 were low, and the latent gene EBNA2 and the lysed genes BZLF1 and gp350/220 were not detected (6). EBNA1, EBNA2, and EBNA3C are the main targets of CD4+T cell response, while EBNA3A, EBNA3B, and EBNA3C are the main targets of CD8+T cell response. However, most of these antigens are not expressed in CAEBV patients, thus helping to evade cellular immunity. The reduced frequency and low expression level of EBNA1 may also contribute to the immune escape mechanism of CAEBV. In addition, studies have shown that mutations in a variety of genes can be found in CAEBV patients, including GATA2, CD27, FANCA, IL2RG, SH2D1A, XIAP, TNFRSF9, PIK3CD, etc (2, 4). The mutations in these genes cause persistent EBV infection. It also seriously affects the function of T and NK cells, resulting in immune dysfunction of the body and ultimately the occurrence of CAEBV (7). Recent research suggests that CAEBV may originate from hematopoietic stem cells infected with EBV (8, 9).

**Figure 2:**
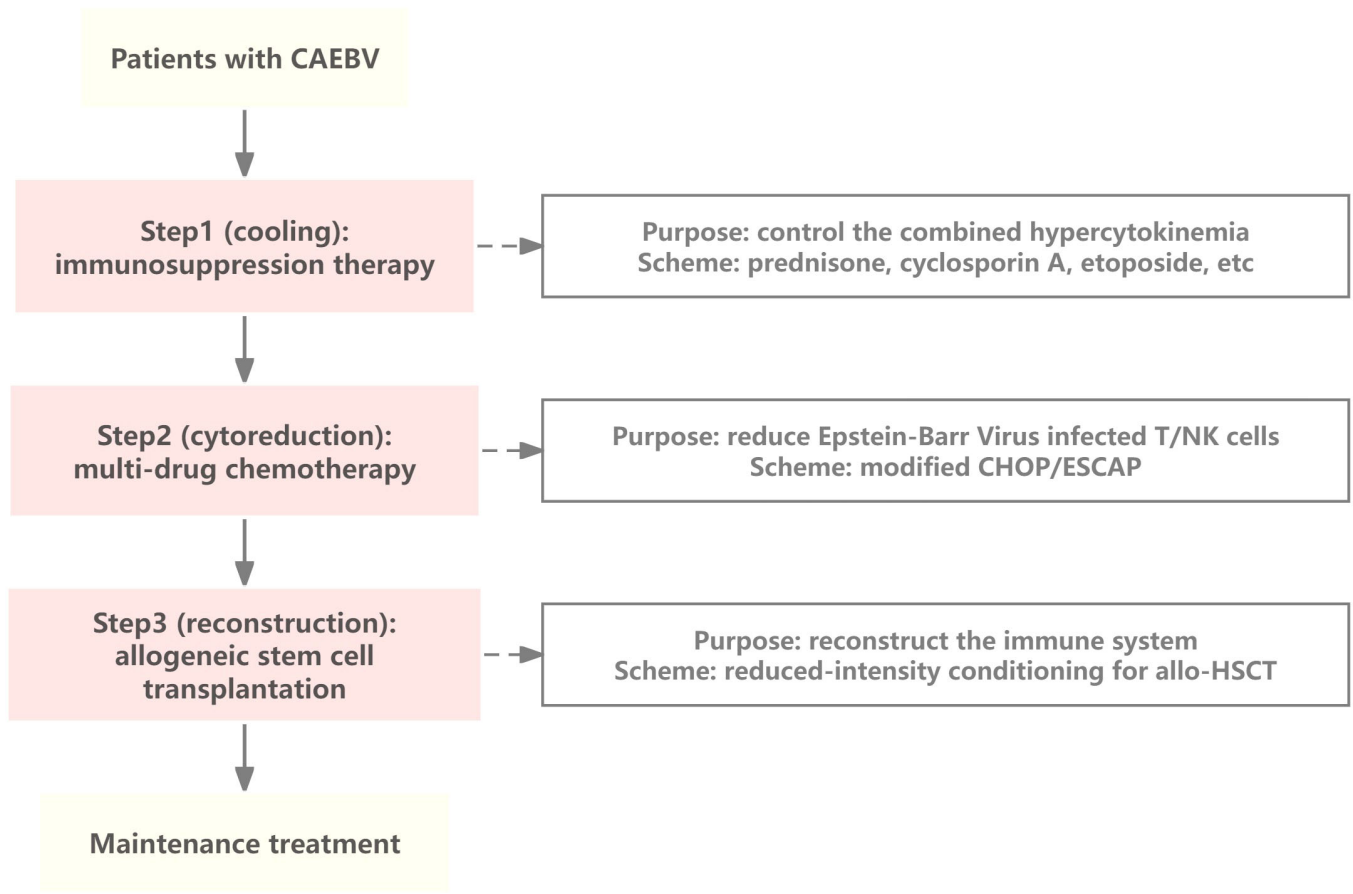
The “three-step” therapy of CAEBV. The first step in the treatment of CAEBV is to control the combined hypercytokinemia by immunosuppressive therapy, the second step is to eliminate EBV-infected T/NK cells as much as possible using combination chemotherapy, and the third step is to achieve reconstitution of immune function by hematopoietic stem cell transplantation. CAEBV, Chronic active Epstein-Barr Virus disease; NK, natural killer; modified CHOP, cyclophosphamide, pirarubicin, vincristine, and prednisolone; ESCAP, etoposide, cytosine arabinoside, L-asparaginase, methylprednisolone, and prednisolone; allo-HSCT, allogeneic hematopoietic stem cell transplantation.

However, a standard therapeutic regimen for CAEBV has not yet been established. The “three-step therapy” proposed by Japanese scholars includes the first step of immunosuppression to control inflammatory response, the second step of clearing EBV-infected lymphocytes through chemotherapy, and the third step of allogeneic hematopoietic stem cell transplantation (allo-HSCT) to achieve immune reconstitution (10) (**Figure 2**). Although allo-HSCT may be the only method to cure CAEBV, the mortality rate of the disease remains high (11).

Given the progress in the application of immunotherapy for CAEBV, new avenues for its treatment have been proposed. This article discusses the application of immunotherapies for CAEBV and summarizes the current progress in immunotherapies for CAEBV.

## 2 Diagnostics of CAEBV

In 2022, Japanese scholars revised the diagnostic criteria for CAEBV, indicating that CAEBV diagnosis requires confirmation of the EBV DNA load and an increase in the number of T/NK cells infected with EBV. The commonly used diagnostic criteria currently include the following: (1) persistent or recurrent IM-like symptoms for ≥ 3 months (>10^2.5^ copies/μg DNA); (2) an increased number of EBV genomes in peripheral blood and/or affected tissues; (3) EBV-infected T or NK cells in peripheral blood and/or affected tissues; (4) chronic illness that cannot be explained by other known disease processes at the time of diagnosis (4). Among them, the main symptoms and signs in the diagnosis of CAEBV include fever, hepatosplenomegalgia, lymphadenopathy, cardiac insufficiency, aneurysm, gastrointestinal symptoms, nervous system symptoms, vasculitis, choroiditis, etc (12). In laboratory diagnosis, high EBV associated antibody titers including anti-viral capsid antigen (VCA)-IgG≥640 and anti-diffuse and restricted early antigen (EA-DR)-IgG≥160 are unique virological feature of CAEBV, but it is not necessary for diagnosis because antibody titers are not elevated in some patients (4).

## 3 Immunotherapies for CAEBV

**Figure 3:**
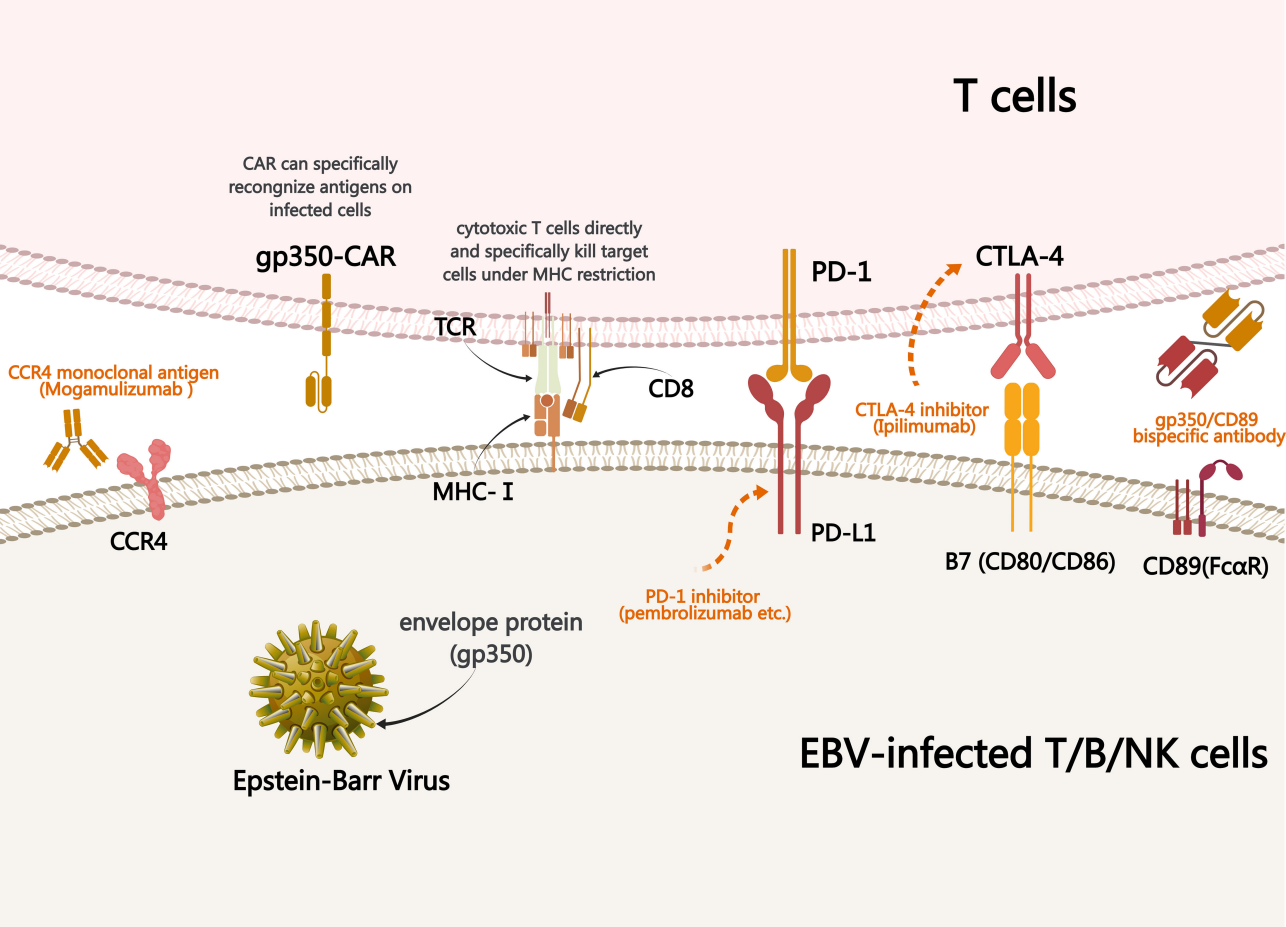
Mechanism underlying treatments used to treat CAEBV. Immunotherapy of CAEBV relies on specific binding between ligands and receptors, antibodies and antigens, resulting in the killing effect of normal immune cells on EBV-infected T/NK cells. PD-1, programmed cell death protein 1; CAR, chimeric antigen receptor; CCR4, cytotoxic T-lymphocyte antigen 4; MHC, major histocompatibility complex; TCR, T cell receptor; CAEBV, chronic active EBV infection.

Currently, there is no standard treatment for CAEBV and allo-HSCT remains the only radical treatment available. Previous studies have shown that antiviral drugs, such as acyclovir and ganciclovir (viral DNA polymerase inhibitors), have limited efficacy against CAEBV (13). Immunotherapy has unique advantages for the treatment of a variety of malignant tumors as it exerts antitumor effect by regulating the immune system of the body. Therefore, it may also be a treatment option for patients with CAEBV, for whom multiple immunotherapy approaches are beneficial (**Figure 3**).

### 3.1 Drug treatments

#### 3.1.1 PD-1 inhibitors

Programmed cell death protein-1 (PD-1) inhibitors are widely used in the immunotherapy of malignant tumors. PD-1 and PD-1 ligand (PD-L1) are also highly expressed in EBV-LPD tissues. In their single-center retrospective study, Ma et al. analyzed 16 patients with CAEBV who received PD-1 inhibitors (two pembrolizumab, nine sintilimab, and five nivolumab) but did not have HLH, and evaluated the efficacy and safety of PD-1 inhibitors (14). The median age of the 16 patients at disease onset was 33 years (range, 11–67 years), with 12 patients responding to PD-1 inhibitors and a median progression-free survival (PFS) of 11.1 months. Three patients achieved clinical complete response (CR) and molecular CR, five achieved and maintained partial response (PR), and four patients transitioned from PR to no response (NR).

Sintilimab is a whole human IgG4 monoclonal antibody that can bind to PD-1 and block its interaction with its ligand. It has been approved for relapsed or refractory classical Hodgkin’s lymphoma (15), and its research on CAEBV is also ongoing. Chen et al. reviewed and analyzed three children receiving sintilimab treatment, including two cases of CAEVB and one case of EBV-HLH (16). After treatment with Sintilimab and an average follow-up of 17.1 months (range 10.0–23.3 months), two patients achieved clinical CR and one achieved clinical PR; the EBV-DNA load in the blood and plasma of all three children decreased by over 50%, indicating the presence of molecular PR. The copy number of EBV DNA in peripheral blood mononuclear cells (PBMCs) is also significantly reduced. The authors of the study believe that the PD-1 inhibitor sintilimab may have significant effects in patients with CAEBV and EBV-HLH. PD-1 inhibitors may restore the body’s immune system and release T cells, providing benefits for patients with CAEBV and EBV-HLH; however, further clinical trials and mechanistic studies are still needed.

Song et al. evaluated the efficacy and safety of sintilimab combined with the immunomodulatory drug lenalidomide in a single-center prospective study (NCT04084626) (17). They included 34 patients, of whom 24 completed at least three courses of treatment. In the evaluation of 24 patients, the overall response rate (ORR) was 54.2% (13/24), the CR rate (CRR) was 45.8%, the PR rate was 8.3%, and the EBV DNA copy number in PBMCs significantly decreased. The median follow-up time was 17.8 months, and 22 patients survived. The median survival was not observed, and the estimated one-year survival rate was 91.3%. Among the 13 effectively treated patients (those who achieved CR and PR), one patient with PR relapsed after maintaining PR status for 4 weeks and received allo-HSCT. The patient ultimately died of allo-HSCT complications; the remaining 12 patients did not receive allo-HSCT, and none of the 12 patients who achieved CR experienced relapse. Among the other 11 patients in whom treatment failed (non-response group), one patient died of disease progression, and one patient ultimately underwent allo-HSCT. These results indicate that the combination of the PD-1 inhibitor sintilimab and lenalidomide is a safe and effective treatment for CAEBV, and the mechanism of action of PD-1 inhibitors in CAEBV may be related to the activation and proliferation recovery of EBV-specific cytotoxic T cells CTLs. However, the sample size included in this study was limited, and the role of lenalidomide in the treatment process was not analyzed; therefore, further exploration is needed.

Nivolumab is a human IgG4 PD-1 antibody that can bind to human PD-1 with high affinity, blocking the interaction between PD-1 and PD-L1 and PD-L2 ligands (18). Dalela et al. included 11 eligible patients in their trial using nivolumab to treat EBV+LPD and EBV+non-Hodgkin lymphoma (NHL), including 6 (55%) with EBV+LPD and 5 (45%) with NHL (19). Among the 10 assessable subjects (one died before re-staging), the ORR was 60% and 50%, respectively. The CRR of both the LPD and NHL queues was 50%. At a median follow-up of 36 months, the 2-year PFS and OS rates were 33% and 72%, respectively. Among them, the 2-year PFS of the LPD queue was 63% and the 2-year OS was 100%. The results of this study indicate that nivolumab is safe and effective for patients with EBV+LPD and is a possible treatment option for EBV+LPD, including CAEBV.

#### 3.1.2 Bispecific antibody

Gp350 is the most important glycoprotein on the EBV surface. CD89 (also known as FcαRI) is expressed constitutively on subsets of monocytes, macrophages, neutrophils, eosinophils, and some dendritic cells. Previous studies have shown that CD89 bispecific antibodies are effective in treating malignant tumors and human immunodeficiency virus (HIV) infection (20). Given this, He et al. used a new bispecific antibody R1-KH, which can bind to gp350 expressed on EBV and EBV-positive tumor cells, as well as CD89 expressed on macrophages and neutrophils (1). The *in vivo* experimental results indicated that R1-KH had a stronger inhibitory effect on EBV than monoclonal antibodies targeting gp350. Researchers created a lymphoma model by injecting the EBV-carrying Raji cells (Burkitt’s lymphoma cell line) into mice and treating them with R1-KH and monoclonal antibodies. The results showed that mice treated with bispecific antibodies had a higher survival rate, confirming that R1-KH was more effective than monoclonal antibodies in inhibiting tumor growth and metastasis of EBV-positive lymphoma cells. Overall, gp350/CD89 bispecific therapy has demonstrated potential in EBV immunotherapy, effectively eliminating EBV and highlighting new directions for EBV-related hematological diseases, including CAEBV.

#### 3.1.3 Others

Mogamulizumab is a monoclonal antibody targeting CC chemokine receptor 4 (CCR4) (21). CCR4 is expressed on the surface of regulatory T cells (Tregs) and is also expressed in most EBV-positive T/NK cell lines. Wilcox et al. showed that mogamulizumab eliminated EBV+T cells in peripheral blood along with CCR4-expressing Tregs, thereby reducing its inhibition of tumor immunity in the body, and had a favorable impact on the tumor immune microenvironment (22). In animal experiments, mogamulizumab induced antibody-dependent cytotoxicity against CCR4-positive cell lines and inhibited the growth of EBV-positive NK cell lymphoma (21). However, CCR4 is also expressed on the surface of type 2 helper T (Th2) cells and skin homing T cells, so the CCR4 antibody Mogamulizumab is currently only approved for cutaneous T cell lymphomas, whose malignant T-cells are skin-homing T-cells and which exhibit a high CCR4-expressing Th2 and Treg phenotypes (21). This suggests that while Mogamulizumab may be an option for the treatment of EBV-associated T/NK LPD, its negative immunomodulation of exposure to Treg production is accompanied by suppression of the positive regulation of humoral immunity by Th2 cells, and thus, the use of Mogamulizumab in the treatment of EBV-associated T/NK LPD still needs to be further explored.

Cytotoxic T-lymphocyte antigen 4 (CTLA-4) is also an immune checkpoint protein that binds with high affinity to B7 ligands on antigen-presenting cells, thereby inhibiting T cell activation signals, reducing cytokine production, and lowering the body’s anti-tumor immune response (23). Ipilimumab, an anti-CTLA-4 monoclonal antibody, has demonstrated positive therapeutic outcomes in multiple malignancies including EBV-positive nasopharyngeal carcinoma, malignant melanoma, lung cancer, and renal cancer (24). Multiple studies are currently underway to evaluate its application in the treatment of hematological diseases.

Antibody-mediated immunogenic epitope delivery to redirect virus-specific CD8+T cells to cancer cells is an emerging and promising therapeutic strategy. Antibody-epitope conjugates (AECs) rely on the proteolytic release of epitopes near the tumor surface, presented by human leukocyte antigen (HLA) class I molecules, to ultimately target and activate virus-specific CD8+T cells to tumor cells. Wulp et al. reported that genetically fused AECs can effectively redirect virus-specific T cells *in vivo* and *in vitro*, significantly reducing tumor growth and increasing overall survival (25) and are a potential treatment option for CAEBV.

Brentuximab vedotin (BV) is an antibody-coupled drug (ADC) targeting CD30, combining the killing effect of conventional chemotherapy with the tumor-targeting properties of antibody drugs. BV is used for refractory EBV-related post-transplant lymphoproliferative disease (PTLD), and significant and persistent clinical efficacy has also been shown in multiple relapsed/refractory EBV-positive and CD30-positive lymphomas. However, there has been no research on its application to CAEBV (26) and further exploration is needed.

### 3.2 Cellular therapies

#### 3.2.1 EBV-specific cytotoxic T cell (EBV-CTL)

Previous studies have shown that infusion of EBV-specific cytotoxic T lymphocytes can effectively restore EBV immunity in patients. Once returned to the patient’s body, it can produce antiviral effects, thereby controlling CAEBV infection.

Miyamura et al. also reported two cases of patients treated with EBV-CTL for CAEBV after HSCT in whom high EBV genomic titers were detected in the peripheral blood at the onset of the disease (27). After transplantation, as the CTL activity increased, the EBV genome titer gradually decreased. These results indicate that an increase in CTL activity may normalize the immune serology of the virus or that enhanced CTL activity can eliminate residual viruses. As early as 2002, studies were using EBV-CTL to treat CAEBV; five patients received EBV-CTL infusion, and four of them were followed up for 6-36 months without relapse. One patient experienced relapse symptoms of fatigue and muscle pain after one year of infusion. Researchers believe that autologous EBV-CTL adoptive immunotherapy may be a safe and feasible alternative treatment for patients with mild/moderate CAEBV infections and that this method should be evaluated in patients with more severe disease forms (28). Meedt et al. reported another patient with persistent CAEBV remission who received third-party EBV-CTL infusion and received allo-HSCT treatment (29). When toxemia persisted in the patient, the infusion of EBV-CTL controlled the symptoms of CAEBV. As host T cells regenerate after transplantation, EBV also undergoes reactivation, which can be controlled by EBV-CTLs from stem cell donors. EBV viremia resolved after the patient first received anti-T-lymphocyte globulin (ATLG) to prevent graft versus host disease (GvHD). EBV-CTL infusion from third-party donors and subsequent allo-HSCT may be an effective and safe treatment for CAEBV, and the combination of EBV-CTL and HSCT brings new hope to patients with CAEBV.

Tablecleucel is the first allogeneic EBV-CTL approved for the treatment of relapsed or refractory EBV-positive PTLD (30). While it has made significant progress in PTLD and EBV toxemia (31), it may also be an effective method for treating CAEBV. However, large-scale clinical trials are currently lacking, and further studies are needed. The Phase II trial ATA129-EBV-205 aimed to evaluate the efficacy and safety of tableclear in EBV-related diseases, including CAEBV. We look forward to the publication of its results (32). Vacciniforme-like lymphoproliferative disorder (HV-LPD) is a manifestation of CAEBV skin infection. Researchers have previously reported a case of HV-LPD treated with cellular immunotherapy (33). They stabilized the disease process using a third-party donor, EBV-CTL, followed by matched unrelated donor HSCT (MUD-HSCT) and subsequently underwent EBV-CTL again. CTL treatment before transplantation did not reduce the EBV viral load, and shortly after transplantation, the EBV viral load could not be detected. To prevent and enhance the anti-EBV effect of transplantation and to reduce the risk of reactivation, patients received multiple cycles of EBV-CTL treatment after transplantation. Currently, the patient is in complete remission, and there has been no recurrence of EBV infection.

Sinha et al. have developed an ‘off-the-shelf’ allogeneic EBV-CTL that can effectively recognize the expression of EBV nuclear antigen1 (EBNA1) and/or latent membrane protein (LMP) 1 and LMP2. *In vitro* experiments can effectively identify EBV-positive tumor cells. Various tumor models have demonstrated efficacy and safety. In addition, researchers have overcome drug resistance by sequentially infusing two different allogeneic EBV-CTLs with different HLA allele restrictions; the combined use of PD-1 inhibitors (nivolumab) can enhance the efficacy of EBV-CTL (34). Their research provided new ideas for the application of EBV-CTLs in the treatment of CAEBV.

BamH1 A rightward open-reading frame-1 (BARF1) is a secreted protein that plays an important role in the malignant transformation of EBV-positive cells and is part of the EBV immune escape strategy. Kalra et al. found that BARF1 is a target of CD4 and CD8-positive T cells, and BARF1-specific T cells can be activated and expanded in the peripheral blood of EBV-positive patients, thereby identifying and killing EBV-positive tumor cells (35). Based on this conclusion, researchers have designed EBV-CTLs targeting BARF1, which are currently undergoing clinical trials for the treatment of EBV-positive lymphoma and nasopharyngeal carcinoma. However, it has not yet been used for the treatment of CAEBV and further research is needed.

Sharma et al. found that the constitutive Interleukin-7 receptor (C7R) can enhance the persistence, amplification, and anti-tumor activity of EBV-specific T cells. They used EBV virus-specific T cells expressing C7R (C7REBVST) to treat xenograft lymphoma mouse models, achieving faster and more durable tumor control. Their research suggested that C7R could enhance the persistence of EBV-CTLs during adoptive transfer. Simultaneously, the level of phosphorylated STAT5 (a downstream molecule in the cytokine signaling axis mediated by the Interleukin-7 receptor) increases, maintaining the antigen-specific and cytotoxic effects of CTL (36). Therefore, C7R-modified EBV-CTL may be a potential method for enhancing the efficacy of EBV-CTL in the treatment of CAEBV.

However, the use of EBV-CTL in CAEBV is still limited. Early studies found that EBV+ T/NK cells in CAEBV patients do not express the most immunogenic EBNA2/3, but rather EBNA1 with LMP, which cannot be recognized and cleared by EBV-CTL, leading to recurrent EBV activity *in vivo*, thus limiting the application of EBV-CTL in CAEBV (37, 38). EBV-CTL targeting EBNA1 and/or LMP developed by Sinhaetal. et al. effectively solved the problem. Currently, multiple clinical trials for the CTL treatment of CAEBV are underway, including NCT01945814, NCT05532826, and NCT05688241. We look forward to the release of additional trial data in the future.

#### 3.2.2 CAR-T

Chimeric antigen receptor T (CAR-T) cells are a rapidly developing cell therapy technique that is widely used in the treatment of B-cell malignancies.

gp350 is a potential viral antigen used in cellular immunotherapy. Studies have shown that CAR-T cells targeting gp350 can be used to treat EBV virus-positive lymphoproliferative diseases. *In vitro* studies showed that gp350CAR-T cells can specifically recognize and kill target cells and control or reduce EBER-positive B-cell lymphoproliferation, tumor progression, and inflammatory responses in animal experiments (39). ZT002 is a CAR-T cell that targets gp350 and exhibits cytotoxic effects on various cell lines, such as nasopharyngeal carcinoma and gastric cancer that are gp350 positive *in vitro*. *In vivo* experiments have also shown dose-dependent therapeutic effects on nasopharyngeal carcinoma (40). Therefore, gp350 may be a target for CAR-T cell treatment of CAEBV and deserves further research. Clinical trials of EBV-specific CAR-T cells (BRG01, NCT05864924) for the treatment of EBV-positive lymphoma are currently underway. Therefore, CAR-T cells may be a future treatment method for CAEBV and their efficacy can be demonstrated after clinical transformation.

#### 3.2.3 TCR-T

The presence of LMP2A in EBV limits the expression of EBV genes while inhibiting CTL production, thus avoiding the destruction of EBV genes by CTL (41). Zhang et al. proposed that LMP2A-specific T cell receptor-engineered T (TCR-T) cells are a promising method for treating EBV-related malignant tumors. They identified and optimized multiple LMP2A reactive TCRs, cloned them into lentiviral vectors, and then transfected them into peripheral T cells, ultimately demonstrating the specific recognition of LMP2A presented by autologous dendritic cells and lymphoblasts by these T cells *in vitro* and *in vivo*, providing a new approach for LMP2A specific TCR-T treatment of CAEBV (42).

#### 3.2.4 Others

Tumor-infiltrating lymphocytes (TILs) are used to treat various solid tumors. Currently, it has been used in the treatment of EBV-positive nasopharyngeal carcinoma patients in China and has achieved good efficacy (43). NK cells can kill virus-infected cells as well as tumor cells downregulated by MHC, so they can be used for diseases caused by EBV infection (44, 45). Chimeric antigen receptor NK cells (CAR-NK) can directly recognize target cells in the absence of major histocompatibility complex (MHC) and do not trigger the common side effects of CAR-T cell therapy (46). Cellular immunotherapy is a rapidly evolving technique with an extremely broad scope for treating CAEBV and deserves further exploration.

## 4 Discussion

CAEBV is a rare EBV-related T/NK cell lymphoproliferative disease. Patients with this disease usually experience systemic organ lesions, such as HLH, liver failure, coronary artery aneurysm, etc (4), with high mortality rates and poor prognosis. Currently, there is no standard therapeutic regimen for this disease, antiviral drugs have limited efficacy against CAEBV, and allogeneic hematopoietic stem cell transplantation is considered the only curative method.

Immunotherapies, including drug and cell therapies, are widely used to treat various malignant tumors. Currently, existing data have shown that programmed cell death protein-1 inhibitors (such as sintilimab and ruxolitinib), bispecific antibodies, proteasome inhibitors, and EBV-specific T cells have demonstrated their effectiveness and safety in CAEBV. CAR-T is a rapidly developing cellular immunotherapy that has demonstrated its unique advantages in malignant tumors derived from B cell (32), and multiple CAR-T products have been launched. However, further research is required to confirm its application to CAEBV. Overall, the treatment of CAEBV remains a challenge, and further research is required. Multiple studies are underway (**Table 1**), resulting in infinite possibilities for treating CAEBV infections.

**Table 1:** Immunotherapy-related clinical trials of CAEBV.

Therapy	Trails	Status	Objects	Drugs
PD-1 inhibitor	NCT04518982	Unknown status	Patients with CAEBV	Sintilimab and lenalidomide
	NCT04084626	Unknown status	Patients with EBV-HLH/CAEBV	PD-1 antibody and lenalidomide
	NCT05039580	Unknown status	Patients with EBV-HLH/CAEBV	PD-1 Monoclonal Antibody
	NCT04690036	Unknown status	Reactivation of EBV for Patients with CAEBV and EBV-HLH after transplantation	PD-1 antibody
	NCT05841342	Recruiting	Patients with EBV-HLH/CAEBV	PD-1 antibody
CD20 Monoclonal Antibody	NCT05258136	Enrolling by invitation	Patients with EBV-HLH/CAEBV	CD20 monoclonal antibody
	NCT05384743	Unknown status	Patients with EBV-HLH/CAEBV	Rituximab
EBV-CTL	NCT05532826	Recruiting	Patients with EBV-HLH/CAEBV	Donor EBV-CTL infusion after allogenic hematopoietic stem cell transplantation
	NCT05688241	Not yet recruiting	EBV-Driven Lymphomas/Diseases	EBV-Tscm cytotoxic T cells
	NCT01956084	Active, Not Recruiting	Severe CAEBV	Cytotoxic T cells
	NCT01945814	Active, Not Recruiting	EBV reactivation or infection	Allogeneic multivirus - directed cytotoxic T lymphocytes
Chemotherapy	NCT05347381	Not yet recruiting	CAEBV	Selinexol combined with dexamethasone

All data comes from https://clinicaltrials.gov/.

PD-1, programmed cell death protein 1; CAEBV, chronic active Epstein-Barr Virus infection; EBV-HLH, Epstein-Barr virus-associated hemophagocytic lymphohistiocytosis, EBV-CTL, Epstein-Barr virus specific cytotoxic T lymphocytes.

## References

[1] He H Lei F Huang L Wang K Yang Y Chen L. Immunotherapy of Epstein-Barr virus (EBV) infection and EBV-associated hematological diseases with gp350/CD89-targeted bispecific antibody. BioMed Pharmacother. (2023) 163:114797. doi: 10.1016/j.biopha.2023.114797 37126928

[2] Jin J Mao X Zhang D. A differential diagnosis method for systemic CAEBV and the prospect of EBV-related immune cell markers via flow cytometry. Ann Med. (2024) 56:2329136. doi: 10.1080/07853890.2024.2329136 38502913 PMC10953786

[3] Chen S Wei A Ma H Zhang L Lian H Zhao Y. Clinical features and prognostic factors of children with chronic active Epstein-Barr virus infection: A retrospective analysis of a single center. J Pediatr. (2021) 238:268–274.e2. doi: 10.1016/j.jpeds.2021.07.009 34260897

[4] Kawada J Ito Y Ohshima K Yamada M Kataoka S Muramatsu H. Updated guidelines for chronic active Epstein–Barr virus disease. Int J Hematol. (2023) 118:568–76. doi: 10.1007/s12185-023-03660-5 PMC10615970 37728704

[5] Fujiwara S Nakamura H. Chronic active Epstein–Barr virus infection: is it immunodeficiency, Malignancy, or both? Cancers. (2020) 12:3202. doi: 10.3390/cancers12113202 33143184 PMC7692233

[6] Iwata S Wada K Tobita S Gotoh K Ito Y Demachi-Okamura A. Quantitative analysis of Epstein-Barr virus (EBV)-related gene expression in patients with chronic active EBV infection. J Gen Virol. (2010) 91:42–50. doi: 10.1099/vir.0.013482-0 19793909

[7] Rodriguez R Fournier B Cordeiro DJ Winter S Izawa K Martin E. Concomitant *PIK3CD* and *TNFRSF9* deficiencies cause chronic active Epstein-Barr virus infection of T cells. J Exp Med. (2019) 216:2800–18. doi: 10.1084/jem.20190678 PMC6888974 31537641

[8] Syrykh C Péricart S Lamaison C Escudié F Brousset P Laurent C. Epstein–Barr virus-associated T- and NK-cell lymphoproliferative diseases: A review of clinical and pathological features. Cancers. (2021) 13:3315. doi: 10.3390/cancers13133315 34282778 PMC8268319

[9] Wang J Su M Wei N Yan H Zhang J Gong Y. Chronic active Epstein-Barr virus disease originates from infected hematopoietic stem cells. Blood. (2024) 143:32–41. doi: 10.1182/blood.2023021074 37824804

[10] Sawada A Inoue M Kawa K. How we treat chronic active Epstein–Barr virus infection. Int J Hematol. (2017) 105:406–18. doi: 10.1007/s12185-017-2192-6 28210942

[11] Dávila Saldaña BJ John T Bonifant C Buchbinder D Chandra S Chandrakasan S. High risk of relapsed disease in patients with NK/T-cell chronic active Epstein-Barr virus disease outside of Asia. Blood Adv. (2022) 6:452–9. doi: 10.1182/bloodadvances.2021005291 PMC8791566 34670275

[12] Arai A. Chronic active Epstein–Barr virus infection: the elucidation of the pathophysiology and the development of therapeutic methods. Microorganisms. (2021) 9:180. doi: 10.3390/microorganisms9010180 33467742 PMC7829705

[13] Bollard CM Cohen JI. How I treat T-cell chronic active Epstein-Barr virus disease. Blood. (2018) 131:2899–905. doi: 10.1182/blood-2018-03-785931 PMC6024635 29712633

[14] Ma Y Zhang P Bao Y Luo H Wang J Huang L. Outcomes of programmed death protein-1 inhibitors treatment of chronic active Epstein Barr virus infection: A single center retrospective analysis. Front Immunol. (2023) 14:1093719. doi: 10.3389/fimmu.2023.1093719 36969150 PMC10036359

[15] Zhang L Mai W Jiang W Geng Q. Sintilimab: A promising anti-tumor PD-1 antibody. Front Oncol. (2020) 10:594558. doi: 10.3389/fonc.2020.594558 33324564 PMC7726413

[16] Chen R Lin Q Zhu Y Shen Y Xu Q Tang H. Sintilimab treatment for chronic active Epstein–Barr virus infection and Epstein–Barr virus-associated hemophagocytic lymphohistiocytosis in children. Orphanet J Rare Dis. (2023) 18:297. doi: 10.1186/s13023-023-02861-9 37736751 PMC10514962

[17] Song Y Wang J Wang Y Wu L You Y Song D. PD-1 blockade and lenalidomide combination therapy for chronic active Epstein-Barr virus infection. Clin Microbiol Infect. (2023) 29:796.e7–796.e13. doi: 10.1016/j.cmi.2023.01.017 36702399

[18] Wang C Thudium KB Han M Wang XT Huang H Feingersh D. *In vitro* characterization of the anti-PD-1 antibody nivolumab, BMS-936558, and *in vivo* toxicology in non-human primates. Cancer Immunol Res. (2014) 2:846–56. doi: 10.1158/2326-6066.CIR-14-0040 24872026

[19] Dalela D Lakhotia R Pittaluga S Muppidi J Steinberg S Phelan J. IBCL-297 phase 2 study of nivolumab in Epstein-Barr virus (EBV)-positive lymphoproliferative disorders (LPD) and EBV-positive non-Hodgkin lymphomas (NHL). Clin Lymphoma Myeloma Leuk. (2023) 23:S451. doi: 10.1016/S2152-2650(23)01356-3

[20] Li B Xu L Pi C Yin Y Xie K Tao F. CD89-mediated recruitment of macrophages via a bispecific antibody enhances anti-tumor efficacy. OncoImmunology. (2018) 7:e1380142. doi: 10.1080/2162402X.2017.1380142 PMC5739557 29296544

[21] Kanazawa T Hiramatsu Y Iwata S Siddiquey M Sato Y Suzuki M. Anti-CCR4 monoclonal antibody mogamulizumab for the treatment of EBV-associated T- and NK-cell lymphoproliferative diseases. Clin Cancer Res. (2014) 20:5075–84. doi: 10.1158/1078-0432.CCR-14-0580 25117294

[22] Wilcox RA. Mogamulizumab: 2 birds, 1 stone. Blood. (2015) 125:1847–8. doi: 10.1182/blood-2015-02-625251 25792728

[23] Lv K Yin T Yu M Chen Z Zhou Y Li F. Treatment advances in EBV related lymphoproliferative diseases. Front Oncol. (2022) 12:838817. doi: 10.3389/fonc.2022.838817 35515118 PMC9063483

[24] Lim DWT Kao HF Suteja L Li CH Quah HS Tan DS. Clinical efficacy and biomarker analysis of dual PD-1/CTLA-4 blockade in recurrent/metastatic EBV-associated nasopharyngeal carcinoma. Nat Commun. (2023) 14:2781. doi: 10.1038/s41467-023-38407-7 37188668 PMC10184620

[25] Van Der Wulp W Remst DFG Kester MGD Hagedoorn RS Parren PWHI van Kasteren SI. Antibody-mediated delivery of viral epitopes to redirect EBV-specific CD8+ T-cell immunity towards cancer cells. Cancer Gene Ther. (2024) 31:58–68. doi: 10.1038/s41417-023-00681-4 37945970 PMC10794138

[26] Kim M Lee JO Koh J Kim TM Lee JY Jeon YK. A phase II study of brentuximab vedotin in patients with relapsed or refractory Epstein-Barr virus-positive and CD30-positive lymphomas. Haematologica. (2021) 106:2277–80. doi: 10.3324/haematol.2021.278301 PMC8327727 33792222

[27] Miyamura T Chayama K Wada T Yamaguchi K Yamashita N Ishida T. Two cases of chronic active Epstein–Barr virus infection in which EBV-specific cytotoxic T lymphocyte was induced after allogeneic bone marrow transplantation. Pediatr Transpl. (2008) 12:588–92. doi: 10.1111/j.1399-3046.2007.00873.x 18266798

[28] Savoldo B Huls MH Liu Z Okamura T Volk HD Reinke P. Autologous Epstein-Barr virus (EBV)–specific cytotoxic T cells for the treatment of persistent active EBV infection. Blood. (2002) 100:4059–66. doi: 10.1182/blood-2002-01-0039 12393655

[29] Meedt E Weber D Bonifacius A Eiz-Vesper B Maecker-Kolhoff B Delecluse S. Chronic Active Epstein-Barr Virus Infection controlled by T allogeneic stem cell transplantation and EBV-specific T-cells. doi: 10.1093/cid/ciad131 36883586

[30] Mahadeo KM Baiocchi R Beitinjaneh A Chaganti S Choquet S Dierickx D. Tabelecleucel for allogeneic haematopoietic stem-cell or solid organ transplant recipients with Epstein–Barr virus-positive post-transplant lymphoproliferative disease after failure of rituximab or rituximab and chemotherapy (ALLELE): a phase 3, multicentre, open-label trial. Lancet Oncol. (2024) 25:376–87. doi: 10.1016/S1470-2045(23)00649-6 38309282

[31] Torno L Dahlberg A Ghobadi A Stiff P Reshef R Weng W. Clinical experience of tabelecleucel in patients with life-threatening complications of Epstein-Barr virus viremia. Blood. (2020) 136:7–8. doi: 10.1182/blood-2020-136052

[32] Prockop S Dinavahi R Navarro W Guzman-Becerra N Sun Y Gamelin L. A multicenter, multicohort, open-label, single-arm per cohort, phase II study to assess the efficacy and safety of tabelecleucel in patients with EBV-associated diseases using an adaptive two-stage study design. Blood. (2020) 136:4–5. doi: 10.1182/blood-2020-136075 32614961

[33] Grześk E Kołtan S Dąbrowska A Urbańczyk A Małdyk J Małkowski B. Case report: Cellular therapy for hydroa vacciniforme-like lymphoproliferative disorder in pediatric common variable immunodeficiency with chronic active Epstein-Barr virus infection. Front Immunol. (2022) 13:915986. doi: 10.3389/fimmu.2022.915986 35990691 PMC9390486

[34] Sinha D Srihari S Beckett K Le Texier L Solomon M Panikkar A. ‘Off-the-shelf’ allogeneic antigen-specific adoptive T-cell therapy for the treatment of multiple EBV-associated Malignancies. J Immunother Cancer. (2021) 9(2):e001608. doi: 10.1136/jitc-2020-001608 33589524 PMC7887372

[35] Kalra M Gerdemann U Luu JD Ngo MC Leen AM Louis CU. Epstein-Barr Virus (EBV)-derived BARF1 encodes CD4- and CD8-restricted epitopes as targets for T-cell immunotherapy. Cytotherapy. (2019) 21:212–23. doi: 10.1016/j.jcyt.2018.08.001 PMC6435433 30396848

[36] Sharma S Sauer T Omer BA Shum T Rollins LA Rooney CM. Constitutive interleukin-7 cytokine signaling enhances the persistence of Epstein–Barr virus-specific T-cells. Int J Mol Sci. (2023) 24:15806. doi: 10.3390/ijms242115806 37958791 PMC10649234

[37] McAulay KA Haque T Urquhart G Bellamy C Guiretti D Crawford DH. Epitope specificity and clonality of EBV-specific CTLs used to treat posttransplant lymphoproliferative disease1. J Immunol. (2009) 182:3892–901. doi: 10.4049/jimmunol.0803572 19265169

[38] Kimura H Hoshino Y Hara S Sugaya N Kawada J Shibata Y. Differences between T cell-type and natural killer cell-type chronic active Epstein-Barr virus infection. J Infect Dis. (2005) 191:531–9. doi: 10.1086/427239 15655776

[39] Slabik C Kalbarczyk M Danisch S Zeidler R Klawonn F Volk V. CAR-T cells targeting Epstein-Barr virus gp350 validated in a humanized mouse model of EBV infection and lymphoproliferative disease. Mol Ther Oncol. (2020) 18:504–24. doi: 10.1016/j.omto.2020.08.005 PMC7479496 32953984

[40] Zhang X Wang T Zhu X Lu Y Li M Huang Z. GMP development and preclinical validation of CAR-T cells targeting a lytic EBV antigen for therapy of EBV-associated Malignancies. Front Immunol. (2023) 14:1103695. doi: 10.3389/fimmu.2023.1103695 36817460 PMC9932894

[41] Rechsteiner MP Bernasconi M Berger C Nadal D. Role of latent membrane protein 2 isoforms in Epstein-Barr virus latency. Trends Microbiol. (2008) 16:520–7. doi: 10.1016/j.tim.2008.08.007 18835714

[42] Zhang C Tan Q Li S Shen L Zhang J Liu Y. Induction of EBV latent membrane protein-2A (LMP2A)-specific T cells and construction of individualized TCR-engineered T cells for EBV-associated Malignancies. J Immunother Cancer. (2021) 9:e002516. doi: 10.1136/jitc-2021-002516 34210819 PMC8252876

[43] Liang YJ Chen QY Xu JX Liu XF Xia JC Liu LT. A phase II randomised controlled trial of adjuvant tumour-infiltrating lymphocytes for pretreatment Epstein-Barr virus DNA-selected high-risk nasopharyngeal carcinoma patients. Eur J Cancer. (2023) 191:112965. doi: 10.1016/j.ejca.2023.112965 37540921

[44] Wang DR Wu XL Sun YL. Therapeutic targets and biomarkers of tumor immunotherapy: response versus non-response. Signal Transduct Target Ther. (2022) 7:331. doi: 10.1038/s41392-022-01136-2 36123348 PMC9485144

[45] Hislop AD Taylor GS Sauce D Rickinson AB. Cellular responses to viral infection in humans: lessons from Epstein-Barr virus. Annu Rev Immunol. (2007) 25:587–617. doi: 10.1146/annurev.immunol.25.022106.141553 17378764

[46] Kim SJ Yoon SE Kim WS. Current challenges in chimeric antigen receptor T-cell therapy in patients with B-cell lymphoid Malignancies. Ann Lab Med. (2024) 44:210–21. doi: 10.3343/alm.2023.0388 PMC10813822 38205527

